# Microbiome-targeted strategies for Alzheimer’s disease: multi-omics signatures, mechanistic links, and intervention evidence

**DOI:** 10.3389/fmicb.2026.1828118

**Published:** 2026-10-28

**Authors:** Xuyang Huang, Xinyue Zhang, Qi Zhu

**Affiliations:** 1 Department of Neurology, Central Hospital Affiliated to Shenyang Medical College, Shenyang, China; 2 Department of Pediatrics, Liaoning Provincial People’s Hospital, Shenyang, China; 3 Department of Emergency, Central Hospital Affiliated to Shenyang Medical College, Shenyang, China

**Keywords:** Alzheimer’s disease, bile acids, gut microbiome, metabolomics, microbiome-targeted intervention, multi-omics, short-chain fatty acids, shotgun metagenomics

## Abstract

Alzheimer’s disease (AD) is increasingly studied in relation to gut microbial mechanisms because microbial metabolism, mucosal barrier function, and peripheral immune activation may influence neuroinflammation and host metabolic stress. This review evaluates microbiome-linked evidence in AD with emphasis on studies that combine metagenomics, metabolomics, inflammatory markers, neuroimaging, cerebrospinal fluid or blood biomarkers, and cognitive phenotyping. Current data support several recurring but not yet causal axes, including bile acid transformation, short-chain fatty acid (SCFA) production, trimethylamine N-oxide (TMAO), bacterial product-related inflammatory signaling, and barrier dysfunction. We also review microbiome-targeted interventions, including diet and prebiotics, strain-specified probiotics and synbiotics, oligosaccharide-based agents, gut-restricted antimicrobials, and fecal microbiota transplantation (FMT). The strength of evidence differs substantially across these strategies: sodium oligomannate has been tested in a phase 3 trial, whereas many probiotic, antimicrobial, and FMT approaches remain early clinical, preclinical, or mechanism-generating. Future trials should define *a priori* microbial, metabolite, immune, and brain biomarker endpoints; control diet, medication, and comorbidity confounding; and validate candidate signatures across independent cohorts before using microbiome profiles for screening or treatment selection.

## 1 Introduction

Dementia is a major global health burden, and Alzheimer’s disease (AD) accounts for most cases. The burden is increasing as populations age; epidemiological estimates indicate tens of millions of affected individuals worldwide and a continuing rise in incident cases (Lanctot et al., 2024). Although amyloid- and tau-targeted therapies have renewed interest in disease modification, AD remains clinically heterogeneous. This has increased attention to peripheral biological processes that may contribute to risk stratification, comorbidity effects, and treatment-response variability.

The gut microbiome is relevant to this discussion because microbial communities participate in nutrient processing, bile acid transformation, mucosal immune signaling, epithelial barrier maintenance, and production of metabolites that can enter host circulation (Chandra et al., 2023). Human studies have reported differences in gut microbial composition and predicted function among cognitively unimpaired individuals, patients with mild cognitive impairment (MCI), and patients with AD dementia. Importantly, some microbiome features have been associated with amyloid and tau measures in cognitively unimpaired older adults, suggesting that gut-related signals may occur before dementia rather than only as a late consequence of advanced disease (Ferreiro et al., 2023).

Taxonomic differences alone do not establish biological relevance. Their interpretation is stronger when they are linked to measurable molecular outputs, such as bile acid metabolism, short-chain fatty acid (SCFA) levels, aromatic amino acid metabolism, trimethylamine N-oxide (TMAO), lipopolysaccharide-related inflammatory activity, or barrier-related immune markers. Multi-omics studies can therefore test whether microbial composition and genes are associated with metabolites, inflammatory mediators, neuroimaging phenotypes, and cognition within the same individuals (Zhao et al., 2025).

Among the candidate pathways, bile acid metabolism has been associated with cognitive impairment and AD-related biomarker profiles, supporting a gut-liver-brain metabolic hypothesis (Nho et al., 2019). TMAO has been detected at higher levels in AD-related cohorts and has been linked to cerebrospinal fluid (CSF) markers of amyloid, tau, and neuronal injury, although these associations do not prove directionality (Vogt et al., 2018). Inflammatory measures related to microbial products, including lipopolysaccharide-binding protein, also show associations with cognitive and clinical features in selected populations (Romo et al., 2024). These findings support mechanistic follow-up but remain vulnerable to confounding by diet, medication exposure, frailty, comorbid disease, and reverse causation.

This review examines microbiome-related evidence in AD by separating three questions that are often conflated: which microbial and metabolite signatures are repeatedly observed; which mechanisms have evidence linking gut-derived signals to neuroinflammation, amyloid or tau biology, vascular dysfunction, and cognition; and which interventions have human evidence rather than only preclinical or indirect support. We focus on multi-omics studies and microbiome-targeted interventions, and we explicitly distinguish biomarker-linked association, mechanistic support, and clinical efficacy. These relationships are summarized schematically in Figure 1.

**Figure 1:**
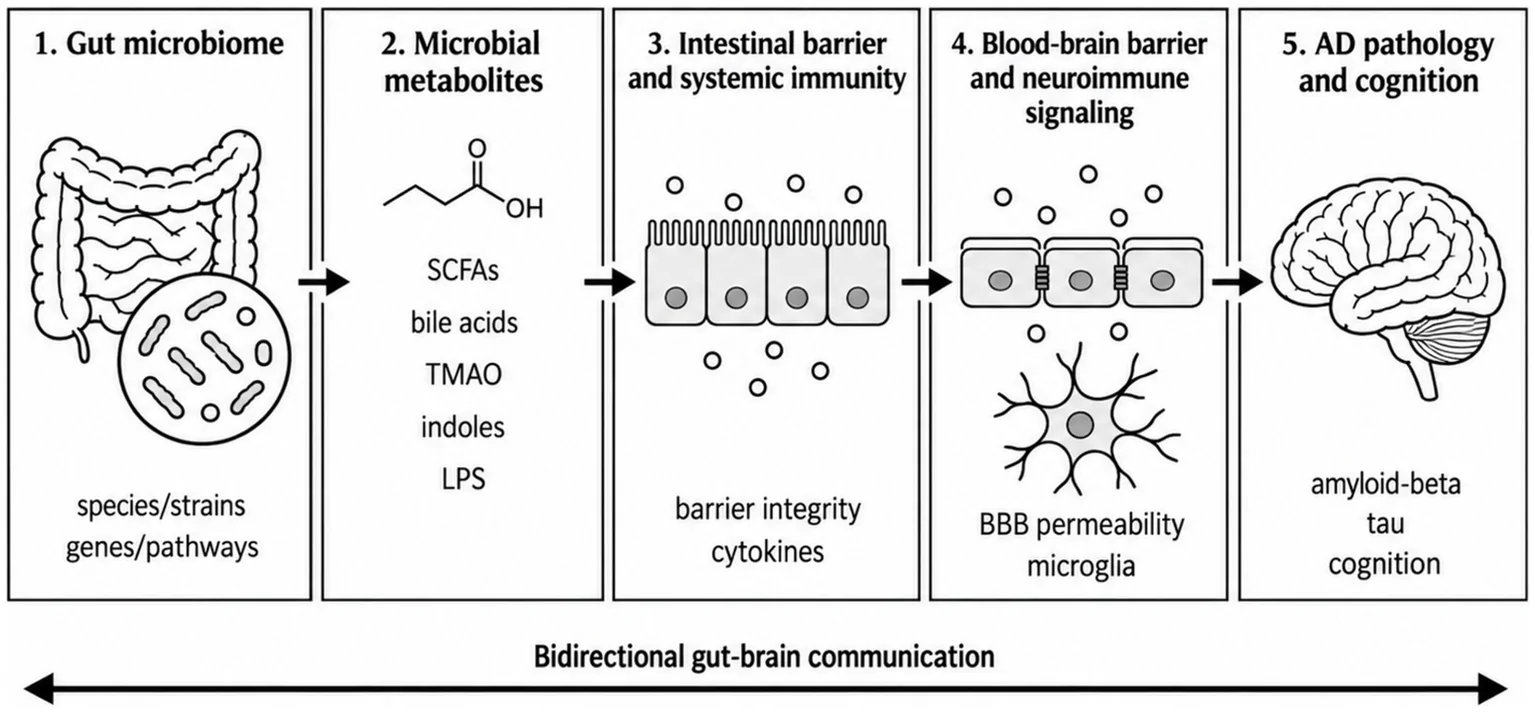
Simplified gut-brain framework linking microbiome-related signals to Alzheimer’s disease. Microbial composition and function are considered together with microbe-related metabolites and products, intestinal barrier and systemic immune signals, blood–brain barrier (BBB) integrity, neuroimmune responses, and AD-relevant pathological and cognitive outcomes. The arrows indicate a conceptual framework and should not be interpreted as proof of a unidirectional causal sequence.

## 2 Multi-omics signatures of the gut microbiome relevant to Alzheimer’s disease

The gut microbiome provides several distinct but interdependent readouts relevant to Alzheimer’s disease (AD): community composition, species-level structure, microbial gene content, fecal and circulating metabolite output, and host inflammatory or barrier-related responses. The most informative human studies are those that align at least one microbial data type with an AD-relevant phenotype, such as cerebrospinal fluid (CSF) amyloid beta (Aβ) and tau measures, amyloid or tau positron emission tomography (PET), plasma phosphorylated tau, magnetic resonance imaging (MRI), or longitudinal cognitive assessment. This design is important because a taxonomic difference alone cannot show whether the microbial community has generated a host-exposed metabolite or immune signal that is relevant to AD pathophysiology (Table 1) (Ferreiro et al., 2023; Zhao et al., 2025; Kang et al., 2025; Sheng et al., 2022; Verhaar et al., 2021; Hosseinkhani et al., 2025).

**Table 1:** Representative multi-omics and biomarker-linked gut microbiome studies relevant to Alzheimer’s disease.

Study / design	Population and stage	Omics data type and specimen	Key microbial or metabolite signal	AD-linked phenotyping	Main contribution and key limitation	Refs
Kang et al.; discovery plus validation metagenomic analysis	AD dementia versus cognitively unimpaired participants; larger validation cohort	Shallow shotgun metagenomics; stool	AD-associated compositional and functional differences; Bacteroidetes/*Bacteroides*-related enrichment and Firmicutes-related depletion were reported in the main analysis; species-anchored pathway signals were examined	CSF Aβ42/Aβ40, p-tau181, t-tau, neurofilament light, neurogranin, soluble TREM2, and α-synuclein	Moves beyond taxonomy by linking species and functional pathways with CSF endophenotypes; limited by small AD dementia subgroup, cross-sectional design, and asynchronous biospecimen/biomarker timing	Kang et al. (2025)
Ferreiro et al.; biomarker-defined preclinical cohort	Cognitively unimpaired older adults with preclinical AD biomarker stratification	Shotgun metagenomics; stool	Microbiome composition differed by preclinical AD status before overt cognitive impairment	Amyloid and tau biomarker framework in cognitively unimpaired participants	Supports early-window microbiome association; does not establish directionality or therapeutic responsiveness	Ferreiro et al. (2023)
Sheng et al.; Sino Longitudinal Study on Cognitive Decline (SILCODE) cross-sectional analysis	Preclinical AD design using cognitively normal older adults	Gut microbiota profiling; stool plus plasma amyloid measures	Gut microbial features added discriminatory information when combined with plasma Aβ	Plasma Aβ-based preclinical AD classification	Shows feasibility of combining microbiome signals with blood biomarkers; cross-sectional and requires external validation	Sheng et al. (2022)
Vogt et al.; case–control study	Clinically diagnosed AD versus cognitively normal controls	16S rRNA sequencing; stool	Genus-level dysbiosis with altered community structure	Clinical diagnosis with CSF biomarker correlations in a subset/framework	Foundational human dysbiosis evidence; limited by genus-level resolution and small cohort size	Vogt et al. (2017)
Verhaar et al.; AD pathology cohort analysis	Participants with cognitive and AD pathology characterization	Gut microbiota composition; stool	Gut microbiota composition related to AD pathology measures	AD pathology measures including biomarker/imaging context	Links microbiota to pathology rather than diagnosis alone; observational design limits causal interpretation	Verhaar et al. (2021)
Fan et al.; MCI-focused shotgun metagenomic study	119 individuals with MCI and 320 cognitively normal controls	Shotgun metagenomics; stool with taxonomic and functional profiling	Fifty-nine microbial species linked to MCI or AD biomarkers; species within *Bacteroides* and *Ruminococcus* showed non-uniform associations; *Akkermansia muciniphila* was associated with lower amyloid burden	Amyloid PET, plasma p-tau181, APOE genotype, cognition, and neurodegeneration-related measures	Demonstrates why species-level resolution is preferable to genus-level summaries; cross-sectional baseline design cannot prove causality	Fan et al. (2025)
Zhao et al.; integrated multi-omics analysis across continuum	Healthy controls, MCI, and AD dementia	16S rDNA sequencing, fecal untargeted metabolomics, and multimodal MRI	Progressive increases in taxa such as *Staphylococcus* and *Bacillus* and decreases in *Anaerostipes*; 26 fecal metabolites, including arachidonic, adrenic, and lithocholic acids, increased along the HC-MCI-AD continuum	Multimodal MRI, cognitive variables, and clinical stage	Provides microbiota-metabolite-brain-cognition mediation hypotheses; cohort size is modest and mechanisms require validation	Zhao et al. (2025)
Nho et al.; ADNI-linked targeted metabolomics	MCI and AD within large biomarker cohort framework	Targeted serum bile acid metabolomics	Altered bile acid profiles and gut-microbial conversion ratios	ATN biomarkers, neuroimaging, and cognition	Supports gut-liver-brain metabolic link; bile acids are strongly confounded by liver, diet, medication, and metabolic status	Nho et al. (2019)
MahmoudianDehkordi et al.; multicohort metabolomics	Cognitive impairment and AD cohorts	Serum bile acid targeted metabolomics	Bile acid alterations associated with cognitive impairment	Cognitive outcomes and disease status in metabolomics cohorts	Replicates bile acid relevance; observational metabolomics cannot identify microbial source alone	MahmoudianDehkordi et al. (2019)
Varma et al.; metabolomic/transcriptomic/pharmacoepidemiologic study	Population cohort with dementia outcomes	Metabolomics focused on bile acid synthesis and modulation	Bile acid synthesis and modulation linked to dementia-related outcomes	Dementia risk and pathology-related inference	Broadens bile acid evidence beyond cross-sectional AD diagnosis; indirect link to gut microbial transformation	Varma et al. (2021)
Vogt et al.; targeted CSF metabolomics	MCI and AD dementia	Targeted metabolomics; CSF	Elevated trimethylamine N-oxide (TMAO)	CSF Aβ and tau markers plus neuronal injury markers	Places a microbiota-related metabolite in a brain-proximal compartment; cannot determine whether CSF TMAO is causal, compensatory, or confounded	Vogt et al. (2018)
Gao et al.; MCI fecal metabolite study	MCI versus normal controls	Fecal SCFA quantification	Reduced fecal SCFAs in MCI with links to amyloid deposition	Amyloid imaging and cognitive status	Supports SCFAs as early metabolite candidates; fecal abundance does not directly quantify systemic or brain exposure	Gao et al. (2023)
Kuehn et al.; SCFA analysis along AD continuum	Adults across AD biomarker/cognitive continuum	Fecal SCFAs; stool	SCFAs varied by sex and amyloid status and were linked to biomarker/cognitive trajectories	Amyloid/tau status and longitudinal biomarker or cognitive measures	Suggests sex-specific metabolite stratification; needs replication before clinical subtyping	Kuehn et al. (2025)
Romo et al.; inflammatory exposure readout	AD patients stratified by APOE genotype	Lipopolysaccharide-binding protein in plasma fractions	Elevated LBP in APOE3/E3 AD subgroup, with genotype-dependent signal	Clinical Dementia Rating and memory performance	Connects microbial product exposure to clinical status; LBP is not AD-specific and is influenced by infection, metabolism, and gut permeability	Romo et al. (2024)
Hosseinkhani et al.; paired compartment multi-omics	Healthy controls to dementia across cognitive stages	Gut microbiota composition, plasma and fecal metabolites, and inflammatory cytokines	Fecal metabolic changes appeared earlier than plasma changes; microbiota, metabolites, and cytokines tracked cognitive impairment stages	Cognitive staging across the spectrum	Shows value of paired feces/plasma plus immune markers; larger longitudinal validation is needed	Hosseinkhani et al. (2025)
Li et al.; systematic review and meta-analysis	AD spectrum across human microbiome studies	Study-level synthesis of 16S rRNA sequencing findings	Recurrent but heterogeneous gut microbiota changes across studies	Diagnosis/stage as reported in included studies	Clarifies reproducibility limits and heterogeneity; limited by varying sequencing pipelines, geography, diet, and medication control across source studies	Li et al. (2024)

AD, Alzheimer’s disease; ADNI, Alzheimer’s Disease Neuroimaging Initiative; Aβ, amyloid beta; APOE, apolipoprotein E; ATN, amyloid/tau/neurodegeneration; CSF, cerebrospinal fluid; HC, healthy control; LBP, lipopolysaccharide-binding protein; MCI, mild cognitive impairment; MRI, magnetic resonance imaging; PET, positron emission tomography; p-tau181, phosphorylated tau 181; rDNA, ribosomal DNA; rRNA, ribosomal RNA; SCFAs, short-chain fatty acids; SILCODE, Sino Longitudinal Study on Cognitive Decline; TMAO, trimethylamine N-oxide; TREM2, triggering receptor expressed on myeloid cells 2; t-tau, total tau.

Early AD microbiome studies mainly used 16S ribosomal RNA (rRNA) sequencing of stool samples. These studies established that fecal community structure can differ between clinically diagnosed AD, mild cognitive impairment (MCI), and cognitively unimpaired controls, and several reports linked genus-level features with CSF biomarkers or clinical phenotypes (Vogt et al., 2017; Zhuang et al., 2018; Saji et al., 2019; Li et al., 2024). Their value is primarily descriptive: they show that dysbiosis is detectable in human cohorts, but they usually cannot distinguish closely related species, cannot resolve strain-level heterogeneity, and infer function only indirectly. These limitations partly explain why individual taxa reported across cohorts are not fully consistent.

Shotgun metagenomics improves mechanistic interpretation by measuring microbial DNA beyond the 16S locus and by linking taxa to gene families or functional pathways. In the Wisconsin Microbiome in Alzheimer’s Risk Study, shallow shotgun metagenomics showed that AD dementia and cognitively unimpaired groups differed in both taxonomic composition and inferred function, with compositional signals partly replicated in a larger Alzheimer Gut Microbiome Project validation cohort (Kang et al., 2025). The study reported AD-associated enrichment of several Bacteroidetes/Bacteroidota-related features and depletion of several Firmicutes-related features, while functional analyses connected species-anchored pathway differences with CSF biomarkers of amyloid, tau, neurodegeneration, synaptic injury, and glial or inflammatory activation (Kang et al., 2025). The important point is not that one phylum is uniformly harmful or protective, but that species and pathways can be mapped to biomarker-linked endophenotypes.

Species-level analysis is particularly relevant because organisms within the same genus may have opposite associations with AD-related measures. A 2025 MCI-focused shotgun metagenomic study found microbial species signatures associated with amyloid burden, plasma phosphorylated tau 181 (p-tau181), neurodegeneration, and cognition; it also showed that species within genera such as *Bacteroides* and *Ruminococcus* did not behave uniformly, while *Akkermansia muciniphila* was associated with lower amyloid burden (Fan et al., 2025). These findings directly address a weakness of genus-level summaries. Species and gene-family resolution can separate bacteria with different capacities for short-chain fatty acid (SCFA) production, bile acid transformation, lipopolysaccharide biosynthesis, tryptophan metabolism, oxidative-stress handling, and amino-acid metabolism. However, sequencing depth still matters: shallow shotgun sequencing can be useful for broad species and pathway profiling, but low-abundance taxa, strain-level variation, and rare gene families are better assessed with deeper shotgun sequencing or targeted validation.

Metabolomics extends microbiome analysis from genetic potential to biochemical output. Bile acids are among the most reproducible candidate axes. Serum bile acid profiles and ratios that reflect gut microbial conversion have been associated with cognitive impairment, amyloid/tau/neurodegeneration (ATN) biomarker status, neuroimaging measures, and dementia-related outcomes (Nho et al., 2019; MahmoudianDehkordi et al., 2019; Varma et al., 2021). These findings support a gut-liver-brain metabolic hypothesis, but they also require caution because bile acids are strongly influenced by diet, liver function, medication exposure, and metabolic comorbidity. Trimethylamine N-oxide (TMAO), a microbiota-related choline and carnitine metabolite, has been reported at higher levels in CSF from MCI and AD dementia participants and was associated with CSF Aβ, tau, and neuronal injury markers (Vogt et al., 2018). SCFAs form a separate axis: fecal SCFA concentrations have been linked to MCI, amyloid deposition, amyloid/tau status, and longitudinal biomarker or cognitive trajectories (Gao et al., 2023; Kuehn et al., 2025).

The compartment sampled should be interpreted carefully. Fecal metabolites reflect luminal production and transformation; plasma metabolites approximate systemic exposure after absorption and hepatic processing; CSF metabolites provide closer proximity to the central nervous system but may not identify the microbial source. Paired-compartment designs are therefore more informative than single-sample studies. A recent plasma and feces multi-omics analysis across cognitive stages reported that fecal metabolic changes may appear earlier than plasma changes and that microbiota, metabolites, and inflammatory cytokines jointly track cognitive impairment stages (Hosseinkhani et al., 2025). Such findings support the use of multi-compartment sampling, while also underscoring the need for larger longitudinal cohorts before these profiles are treated as diagnostic tests.

Inflammatory and barrier-related measures provide an additional interpretive dimension. Lipopolysaccharide-binding protein (LBP) has been reported to differ in AD-relevant subgroups and to correlate with Clinical Dementia Rating and memory performance in an apolipoprotein E (APOE) genotype-dependent context (Romo et al., 2024). These signals are useful because they connect microbial products to host immune exposure, but they are not specific to AD and can be affected by infection, metabolic disease, and intestinal permeability. In this review, precision subtyping refers to the identification of patient subgroups whose microbiome and metabolite profiles correspond to amyloid or tau status, sex-specific metabolite patterns, inflammatory tone, APOE background, or rate of cognitive decline. At present, this should be treated as a stratification hypothesis rather than a validated clinical classification system.

Overall, multi-omics studies have increased biological resolution beyond taxonomic comparisons, but no microbiome biomarker is currently validated for clinical use in AD. At present, these data are most useful for identifying microbial species, functions, metabolites, and inflammatory signals for prospective validation, including intervention studies that measure microbiome, metabolic, inflammatory, and neurotrophic outcomes together (Marizzoni et al., 2026). Future studies should prespecify controls for diet, medication, bowel habit, comorbidity, and sampling time; include external validation; and test whether microbial features change with AD-related biomarkers longitudinally rather than relying on cross-sectional classification.

## 3 Evidence for microbiome-targeted interventions in Alzheimer’s disease

Intervention studies should be compared according to the type and strength of evidence available. Here, we distinguish five categories: randomized trials in Alzheimer’s disease (AD); clinical studies in mild cognitive impairment (MCI) or older adults at risk; human biomarker or feasibility studies without definitive efficacy endpoints; preclinical AD-model studies; and indirect microbiome or neuroimmune evidence from non-AD conditions. Changes in microbial composition, metabolites, or inflammatory markers should not be interpreted as evidence of durable cognitive benefit or disease modification unless clinical outcomes support that conclusion. Representative intervention classes and the current maturity of their evidence are summarized in Figure 2.

**Figure 2:**
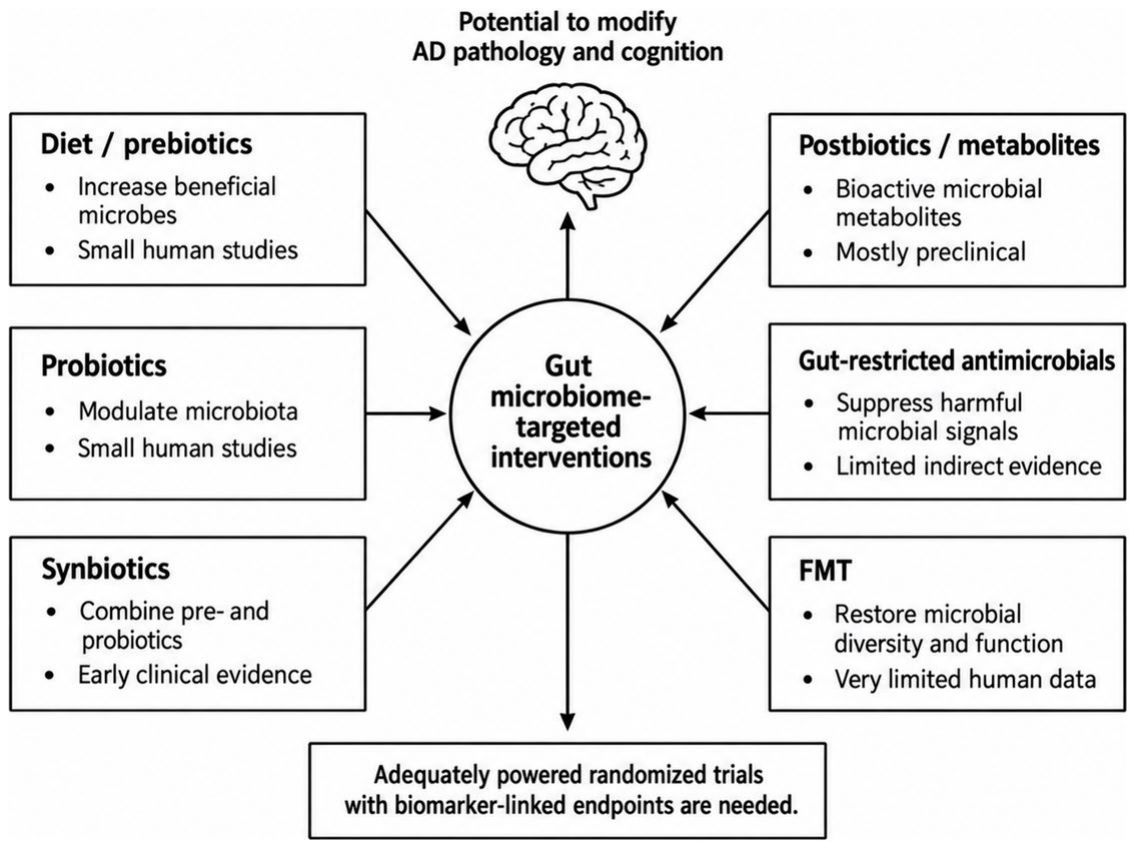
Representative microbiome-targeted intervention strategies in Alzheimer’s disease. The figure summarizes selected intervention classes and the current maturity of their evidence. Human evidence remains limited or heterogeneous for many approaches; therefore, adequately powered randomized studies with predefined microbiome, metabolite, immune, biomarker, safety, and cognitive endpoints are needed. Oligosaccharide-based interventions are discussed separately in Section 3.1.

### 3.1 Oligosaccharide-based interventions: evidence and limitations

Sodium oligomannate (GV-971) has been evaluated in both AD models and a phase 3 AD trial, whereas the other oligosaccharide approaches discussed below remain preclinical. In transgenic models, GV-971 was reported to alter gut microbial composition, reduce peripheral accumulation of selected microbe-associated amino acids, and attenuate neuroinflammatory and amyloid-related readouts together with cognitive improvement (Wang et al., 2019). Subsequent experimental studies have examined microglial and neuroinflammatory responses under GV-971 treatment; these findings support immune modulation as a possible mediator but do not establish a single mechanism of action (Bosch et al., 2024).

The clinical evidence for GV-971 is supported by a 36-week multicenter, randomized, double-blind, placebo-controlled phase 3 trial in mild-to-moderate Alzheimer’s dementia, with the Alzheimer’s Disease Assessment Scale-Cognitive Subscale (ADAS-cog) as the primary endpoint (Xiao et al., 2021). At the same time, the GV-971 experience illustrates the major uncertainty facing microbiome therapeutics: a positive single program does not establish class-wide efficacy. Systematic-review evidence concludes that sodium oligomannate may improve ADAS-cog versus placebo, but that additional larger and higher-quality randomized controlled trials (RCTs), external replication, and longer follow-up are still required to define durability, generalizability, and responder biology (Nourelden et al., 2023).

Other oligosaccharides should be discussed more cautiously because their AD evidence is mostly preclinical or indirect. Chitosan oligosaccharides improved cognition and reduced oxidative stress and inflammatory readouts in an amyloid-beta1-42-induced rat model, suggesting neuroimmune activity but not establishing human efficacy (Jia et al., 2016). Unsaturated mannuronate oligosaccharide has been reported to ameliorate beta-amyloid pathology through autophagy in AD cell models, placing it at the mechanistic cell-model level rather than the clinical level (Bi et al., 2021). Human milk oligosaccharides, especially 2′-fucosyllactose, are relevant because they can modify gut microbial composition, barrier function, vagal signaling, and short-chain fatty acid (SCFA) production; recent AD-mouse evidence links 2′-fucosyllactose to improved cognition through a vagal-afferent and microbiota-metabolite pathway (Jia et al., 2025). These examples broaden the oligosaccharide category but should not be presented as equivalent to GV-971, because their direct human AD evidence is currently absent or insufficient.

### 3.2 Gut-restricted antimicrobials: rationale, evidence, and limitations

Gut-restricted antimicrobials have been proposed as a way to reduce selected microbial or mucosal inflammatory signals while limiting systemic drug exposure. Rifaximin is minimally absorbed and remains concentrated in the intestinal lumen. For AD, the relevant question is whether reducing endotoxin-associated signals, pathobiont activity, or mucosal inflammation produces measurable changes in systemic and brain-related biomarkers. This has not been established as a treatment effect. Antimicrobial exposure may also deplete SCFA-producing bacteria, alter microbial bile acid transformation, and reduce microbiome recovery after treatment, so potential benefit and ecological harm must be evaluated together.

Mechanistically, rifaximin is a gut-selective activator of human pregnane X receptor (PXR) signaling. In PXR-humanized systems, rifaximin induces detoxification and transport programs and dampens inflammatory transcriptional activity (Ma et al., 2007). In intestinal epithelial models and human colon tissue, rifaximin inhibited lipopolysaccharide (LPS)-induced nuclear factor kappa B (NF-κB) activation through a PXR-dependent pathway and reduced inflammatory chemokine and cytokine transcripts (Mencarelli et al., 2011). Complementary epithelial-barrier data support effects on tight-junction integrity and innate signaling; in a Caco-2 toxin challenge model, rifaximin reduced activation of the Toll-like receptor 4 (TLR4)/myeloid differentiation primary response 88 (MyD88)/NF-κB axis and improved barrier-related protein readouts (Esposito et al., 2016). Human observational data in cirrhosis further show that rifaximin can reduce endotoxemia-related indices and modify gut microbiota and permeability-associated biomarkers, which is not AD-specific but demonstrates that a gut-restricted antibiotic can shift systemic exposure to microbial products (Kaji et al., 2020).

An unresolved issue is whether rifaximin-induced microbiome changes preserve beneficial metabolite functions. Because SCFAs and bile acids are candidate pathways in AD microbiome research, antimicrobial studies should measure more than bacterial composition. Fecal and plasma SCFAs, primary-to-secondary bile acid ratios, LPS or LPS-binding protein, inflammatory cytokines, permeability-related markers, and neurodegeneration markers should be assessed within the same protocol. Without these measurements, a decrease in inflammatory markers cannot be separated from possible loss of butyrate-producing taxa or unfavorable changes in bile acid metabolism.

AD-specific rifaximin evidence remains early and exploratory. In a pilot open-label study of probable AD, 3 months of rifaximin treatment was associated with reduced serum neurofilament light and increased fecal Firmicutes, suggesting biological activity but not definitive clinical efficacy (Suhocki et al., 2022). The registered trial framework prespecified cognitive testing, blood-based neuronal and inflammatory markers, and stool microbiome profiling, which is appropriate for an exploratory mechanistic design (U.S. National Library of Medicine, 2019a). Fidaxomicin and other non-absorbable or minimally absorbed antibiotics have also been discussed as experimental means of altering gut microbial composition, but direct AD evidence is absent. Fidaxomicin has gut-localized activity and is relatively narrow-spectrum in the context of *Clostridioides difficile* infection, with studies reporting less disruption of the normal microbiota than broader comparators (Louie et al., 2012; Ajami et al., 2018). Any AD study of fidaxomicin would therefore require careful justification, antimicrobial-stewardship safeguards, and metabolite measurements to determine whether beneficial SCFA and bile-acid profiles are preserved.

### 3.3 Probiotics and synbiotics: clinical and preclinical evidence

Human probiotic studies in AD remain relatively small and heterogeneous. A 12-week double-blind randomized trial in mild-to-moderate AD reported improved Mini-Mental State Examination (MMSE) scores together with changes in insulin resistance and lipid measures (Akbari et al., 2016). However, an expression of concern was later issued for this article, so its findings should be treated as hypothesis-generating rather than definitive evidence (Frontiers Editorial Office, 2020). A separate trial combining probiotics with selenium reported changes in clinical, metabolic, and molecular outcomes, but the co-supplementation design does not allow the probiotic contribution to be isolated (Tamtaji et al., 2019).

Some clinical studies have added inflammatory and neurotrophic biomarkers to cognitive outcomes. In a randomized placebo-controlled study in mild-to-moderate Alzheimer’s dementia, probiotic supplementation was assessed using serum brain-derived neurotrophic factor (BDNF), inflammatory and oxidative-stress markers, and cognition (Hsu et al., 2023). In a 12-week multicenter randomized trial, *Lactobacillus rhamnosus* HA-114 and *Bifidobacterium longum* R0175 were tested separately, with cognitive and anxiety-related outcomes reported versus placebo (Akhgarjand et al., 2022). In suspected MCI, *Bifidobacterium breve* MCC1274 was assessed using cognitive tests together with MRI-derived brain atrophy measures (Asaoka et al., 2022). These studies provide human biomarker data, but they do not establish disease modification.

Animal studies can investigate mechanisms but do not establish clinical efficacy. *Bifidobacterium breve* MCC1274 mitigated AD-like pathological and neuroinflammatory endpoints in mouse models, providing biological support for the MCI trial but not proving disease modification in humans (Asaoka et al., 2022; Abdelhamid et al., 2022). In a 3xTg-AD model under metabolic challenge, the Lab4b consortium was evaluated against cognitive, pathological, and metabolic markers, testing whether probiotic effects are context-dependent when metabolic inflammation is present (Webberley et al., 2022). A human-origin probiotic cocktail study in amyloid precursor protein/presenilin 1 (APP/PS1) mice mapped a gut-to-systemic-to-brain sequence that included reduced hippocampal Aβ burden, suppressed microglial activation and brain cytokines, improved blood–brain barrier (BBB) tight-junction readouts, and reduced systemic inflammation and gut-permeability markers (Prajapati et al., 2025). These findings can help identify candidate mechanisms, but they remain preclinical.

Synbiotics combine a live strain with a fermentable substrate. In a randomized double-blind trial, continuous ingestion of *Bifidobacterium animalis subsp. lactis* GCL2505 with inulin was associated with improved cognitive function and changes in bifidobacteria and inflammatory markers (Azuma et al., 2023). Mechanistic evidence for specific metabolite pathways remains largely preclinical. In a female AD mouse model, *Lactobacillus suilingensis* plus inulin improved cognition and was associated with changes in indole-3-lactic acid metabolism (Yang et al., 2025). Separate experimental work showed that microbiome-derived indole-3-lactic acid reduced amyloidopathy through aryl hydrocarbon receptor signaling in the brain (Kim et al., 2024). These findings support further testing of this pathway but do not establish a clinical synbiotic effect in AD.

### 3.4 Dietary interventions and microbiome-associated biomarker changes

Controlled dietary interventions can test whether microbiome-associated biomarkers are modifiable and whether their changes occur alongside AD-relevant biological measures. A useful design prespecifies the dietary exposure and measures microbial taxa or genes, fecal or plasma metabolites, inflammatory markers, cerebrospinal fluid (CSF) biomarkers, brain physiology, and cognition before and after the intervention. Coordinated change across these measures may strengthen a mechanistic hypothesis, but it does not by itself demonstrate disease modification.

Modified Mediterranean-ketogenic diets alter carbohydrate and fat intake, gut microbial fermentation, and host ketone and lipid metabolism over relatively short periods. In MCI, one study reported changes in gut microbiome features and SCFAs that were associated with CSF AD biomarkers after a modified Mediterranean-ketogenic diet (Nagpal et al., 2019). In a separate pilot crossover study of older adults at risk for AD, the diet was associated with increased CSF Aβ42 and decreased tau, as well as increased cerebral perfusion and cerebral ketone-body uptake in a subsample (Neth et al., 2020). These studies support biomarker measurement within dietary trials but are not evidence of clinical efficacy.

Controlled feeding studies that jointly measure the microbiome, metabolome, and dietary exposures can identify biomarkers that change with diet. In adults at risk for AD, a ketogenic versus low-fat intervention produced coordinated changes in metabolomic, microbiome, and dietary exposure measures (Dilmore et al., 2023). Preclinical dietary evidence should be distinguished from human trials: in a transgenic AD mouse model, a modified Mediterranean-style diet altered gut-microbiome-related measures and AD-relevant neurocognitive outcomes (Park et al., 2024). In humans, the PROMOTe randomized controlled trial reported cognitive improvement after a prebiotic intervention in older adults (Ni Lochlainn et al., 2024). The relevance of these findings to AD-specific disease modification remains uncertain because study populations, interventions, and endpoints differ.

Potential mechanisms linking dietary change to microbiome-associated brain outcomes include changes in SCFA production, gut-barrier integrity, immune regulation, ketone metabolism, and bile acid signaling. Increased fermentable substrate availability can raise SCFA production, which may influence microglial maturation, gut-barrier integrity, regulatory immune tone, and histone deacetylase-dependent transcriptional programs. Ketone metabolism may alter neuronal energy availability and oxidative-stress handling, while bile acid remodeling can influence farnesoid X receptor and Takeda G protein-coupled receptor 5 (TGR5)-related metabolic and immune signaling. These mechanisms are not mutually exclusive and are likely modified by host genotype, baseline microbiome configuration, sex, medication exposure, and vascular-metabolic risk. Future dietary studies should therefore stratify or adjust for apolipoprotein E genotype, antidiabetic and lipid-lowering medications, antibiotic exposure, habitual diet, and baseline amyloid or tau biomarker status.

### 3.5 Fecal microbiota transplantation: experimental evidence and safety considerations

Fecal microbiota transplantation (FMT) directly transfers a donor microbial community and can produce large changes in recipient microbiota and metabolite profiles. Direct human evidence for FMT efficacy in AD remains very limited; current evidence consists mainly of case-level observations and feasibility-oriented trial frameworks rather than adequately powered efficacy trials (Hazan, 2020; U.S. National Library of Medicine, 2019b). Animal studies provide mechanistic evidence that large-scale microbiome perturbation can alter amyloid deposition and neuroinflammatory phenotypes, but these findings do not establish benefit in patients with AD (Minter et al., 2017).

Animal and mechanistic studies have also examined how gut microbial changes affect microglial responses to amyloid. These data suggest that microglial activation and amyloid clearance may be useful mechanistic endpoints when testing microbiome manipulation (Dodiya et al., 2022). Other studies have linked gut dysbiosis with peripheral immune-metabolic signaling and brain pathology, providing additional pathways that can be measured in experimental FMT studies (Chen et al., 2020).

The safety and implementation constraints of FMT are substantial. Potential adverse effects include gastrointestinal symptoms, infection transmission, transfer of antimicrobial-resistant or pathogenic organisms, donor variability, unpredictable engraftment, unintended metabolic effects, and regulatory complexity. U.S. Food and Drug Administration (FDA) safety communications have specifically warned about serious infections and hospitalizations associated with pathogen transmission through investigational FMT products (U.S. Food and Drug Administration, 2020). Donor-to-donor variability and the cost and complexity of screening remain major barriers for non-urgent indications, including neurodegenerative disease (Rasmussen et al., 2024). For AD research, any FMT study should therefore include rigorous donor screening, adverse-event surveillance, antimicrobial-resistance monitoring, donor and recipient multi-omics profiling, and predefined metabolite, immune, and neurodegeneration endpoints; cognition alone would be insufficient.

## 4 Discussion

Human studies show that gut microbial measures can be associated with AD-relevant biomarkers when microbial profiling is combined with metabolites, inflammatory markers, CSF or blood biomarkers, neuroimaging, and cognition. These associations identify candidate gut-liver-brain, gut-immune-brain, and gut-barrier-brain pathways, but they do not establish that microbiome variation initiates or modifies AD pathology. This distinction is important because many reported associations remain cross-sectional and are susceptible to confounding and reverse causation.

Microbiome signatures may be particularly relevant to research in preclinical or prodromal AD rather than as stand-alone diagnostic tests. Stool metagenomics and metabolomics could be evaluated for cohort enrichment or biological subgrouping, but such uses require external validation against amyloid, tau, neurodegeneration, and longitudinal cognitive measures, with strict control of diet, medications, bowel habit, frailty, vascular-metabolic disease, and APOE genotype. Without these controls, microbiome panels may primarily reflect lifestyle and comorbidity rather than AD-specific biology.

Intervention studies should be interpreted according to evidence level and mechanistic support. Sodium oligomannate has phase 3 randomized evidence in AD, but replication, longer follow-up, and responder analyses are still needed. Probiotics, synbiotics, prebiotics, dietary programs, gut-restricted antimicrobials, oligosaccharides, microbial metabolite approaches, and FMT are supported by different combinations of clinical, biomarker, and preclinical evidence. Studies should therefore prespecify a sequence of endpoints from exposure and microbial change to metabolite, immune or barrier measures, AD biomarkers, and clinical outcomes rather than relying on cognition alone.

Several limitations must be addressed before microbiome-targeted approaches can be evaluated for routine clinical use. First, taxonomic findings should be supplemented by species-, gene-, pathway-, and metabolite-resolved analyses. Second, fecal, plasma, and CSF measurements should be interpreted according to the biological compartment sampled. Third, antimicrobial and FMT-based studies require explicit safety monitoring because microbiome disruption or replacement can have unintended metabolic, infectious, and antimicrobial-resistance consequences. Fourth, dietary and probiotic trials should separate general health effects from AD-relevant effects by using biomarker-defined populations and prespecified intermediate endpoints.

Future studies should prioritize longitudinal multi-omics cohorts, intervention trials with embedded mechanistic endpoints, independent validation, and appropriate causal-inference analyses such as Mendelian randomization or mediation analysis when their assumptions are met. Rather than assuming a universal microbiome therapy for AD, clinical studies should test predefined patient subgroups with measurable gut-derived inflammatory or metabolic abnormalities and determine whether those abnormalities change together with brain-relevant biomarkers and clinical outcomes.

## 5 Conclusion

Current evidence shows associations between gut microbiome-related measures and AD heterogeneity, particularly involving bile acid metabolism, SCFAs, TMAO, barrier-related inflammatory signaling, and host-microbe immune interactions. Multi-omics studies have improved biological interpretation by linking microbial taxa and genes with metabolites, inflammatory mediators, imaging or fluid biomarkers, and cognition. Causal evidence remains incomplete, and most microbiome-targeted interventions should be regarded as experimental or subgroup-oriented approaches rather than established disease-modifying therapies. Future studies should emphasize prospective validation, prespecified biomarker endpoints, evidence grading, and safety monitoring, while separating potentially modifiable gut-brain associations from nonspecific effects of ageing, frailty, diet, and comorbidity.
